# Bilateral T-shaped Scrotoplasty as an Ancillary Technique of Staged Anterior Urethroplasty: The End Justifies the Means

**DOI:** 10.7759/cureus.27810

**Published:** 2022-08-09

**Authors:** Luisa Halbe, Bobirjon Ergashev, Axel Heidenreich, Leonidas Karapanos

**Affiliations:** 1 Department of Urology, Uro-Oncology, Robot-Assisted and Reconstructive Surgery, Faculty of Medicine and University Hospital Cologne, University of Cologne, Cologne, DEU; 2 Department of Urology, Andijan State Medical Institute, Andijan, UZB

**Keywords:** urethral reconstruction, scrotal surgery, scrotoplasty, urethral stricture, staged urethroplasty

## Abstract

Staged urethroplasty is performed to treat long-segment obliterating anterior urethral strictures. The technique is particularly challenging when the penobulbar junction is involved, as it requires the transection of the scrotum and the formation of lateral testicular fans. To date, there is no established surgical protocol for this ancillary technique in large volume scrotums with excess skin. We report a case of staged urethroplasty with the necessity of performing T-scrotoplasty due to bulky scrotum. After six months, the T-plasty was successfully resolved, and a new scrotum was formed from the two hemiscrota. In conclusion, scrotoplasty using a bilateral T-plasty is an excellent technique to overcome the necessity of splitting the scrotum by externalizing the urethra to allow for excellent buccal mucosal graft healing over a period of six months. The two separate testicular fans can be reapproximated along the raphe after re-tubularizing the urethra in the 2nd stage, shaping a new scrotum with satisfactory cosmetic results.

## Introduction

The treatment of obliterating, long-segment anterior urethral strictures poses a significant challenge to reconstructive urologists. Depending on the history of prior surgeries and the extent of spongiofibrosis, various surgical methods are available, which must be decided on a case-by-case basis. These include free oral mucosal grafts, pedicled penile skin flaps, and flap/graft combinations [1-3]. A staged approach becomes necessary in cases of extensive spongiofibrosis, as it allows for robust graft healing. During re-tubularization, corrections may be performed in case of recurrence of fibrosis [2]. Usually, staged urethroplasty is performed on the penile urethra for anatomic reasons (thinner corpus spongiosum than bulbar) [4]. However, the technique may involve all segments of the anterior urethra. In the case of penobulbar junction involvement, the additional challenge is to transect and reassemble the scrotum in a way to create two separate testicular compartments lateral to the open neourethral plate. To date, there is no established surgical protocol for this technique in large-volume scrotums with excess skin. The aim of this case report is to present a feasible approach to skin placement as part of a lengthy two-stage urethroplasty for bulky scrotum.

## Case presentation

**Figure 1:**
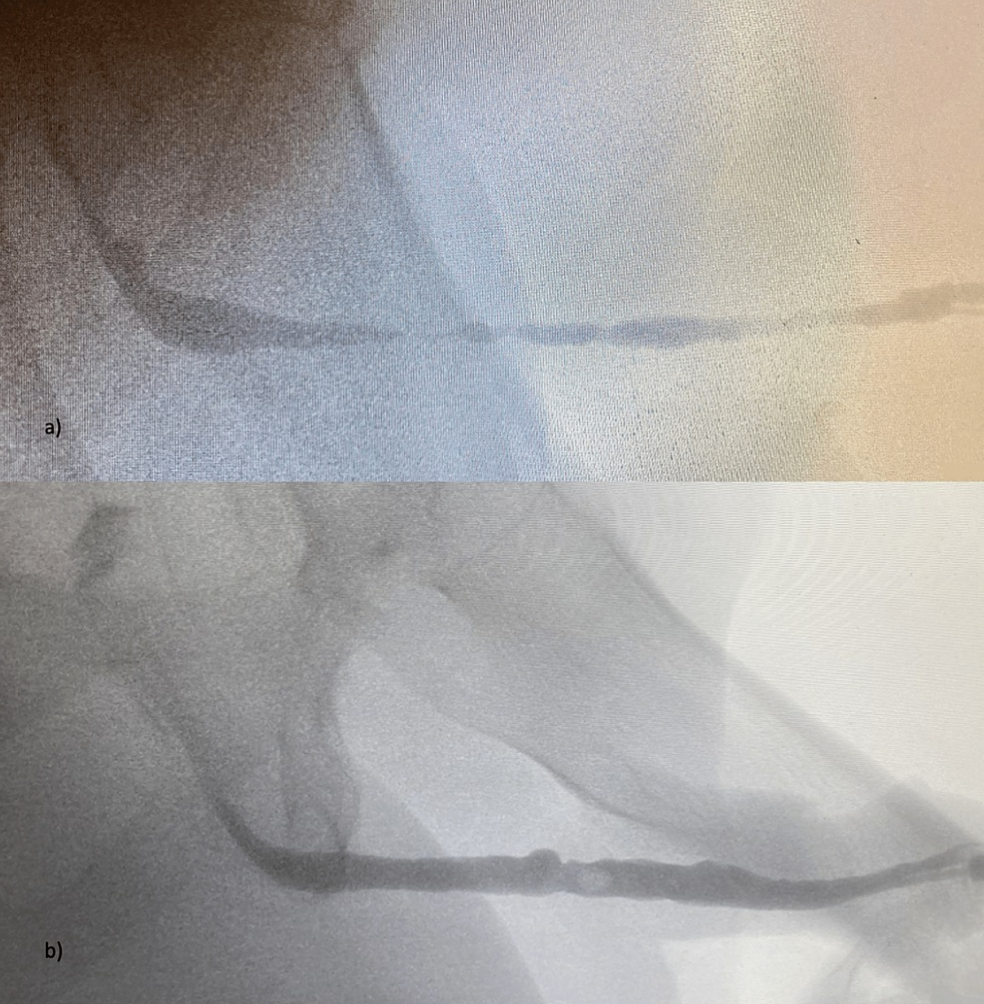
Retrograde urethrogram (RUG) a) Pre-surgery; b) Post-surgery (after 2nd stage)

We report a case of a 66-year-old patient presenting with obstructive low urinary tract symptoms (LUTS) due to a recurrent long-segment penobulbar iatrogenic urethral stricture after two consecutive transurethral bladder tumor resections for low-risk pTa, G2 urothelial carcinoma 10 years prior. To enable regular follow-up cystoscopies, six direct vision internal urethrotomies (DVIU) had been performed in recent years. The patient had no comorbidities and reported satisfactory erectile function with an IIEF-5 (International Index of Erectile Function) score of 23. At the time of initial presentation, urinary diversion via suprapubic catheter was performed following acute urinary retention. The reported IPSS (International Prostate Symptom Score) was 32 with an IPPS-QoL (Quality of Life) of 5 (unhappy). A retrograde urethrogram (RUG) showed a long-segment penobulbar stricture with a nearly obliterated urethral lumen (Figure 1a). Cystoscopy was therefore not possible.

Sonographically, there was marked spongiofibrosis in the entire segment. Due to the extent of the stricture and the multiple prior interventions, we indicated open urethroplasty with oral mucosal augmentation; intraoperatively, a decision should be made regarding a staged procedure if necessary. The surgery was performed by a senior surgeon (LK) with extensive experience in urethroplasty.

1st stage

Using the Kulkarni technique, the penile shaft was mobilized and invaginated down through a perineal incision [5]. A ventral longitudinal midline incision of the corpus spongiosum and urethra was made along the stricture. The distal stricture margin was located 1 cm proximal to the coronary sulcus and the proximal margin in the bulbar urethra. The overall stricture length was 15 cm with severe spongiofibrosis and complete obliteration of the lumen over a distance of 4 cm in the bulbar segment. Based on this finding, the indication for staged technique according to Bracka was given [6].

The penile shaft was brought back orthotopically, and the skin was opened longitudinally over the entire stricture. This involved an incision across the raphe from the coronary sulcus, along the scrotum to the existing perineal access. The scrotum was completely divided down to the urethra into two separate hemiscrota. Before graft implantation, the fibrous portions of the urethral plate were resected. Complete resection of the bulbar obliterated corpus spongiosum was necessary.

**Figure 2:**
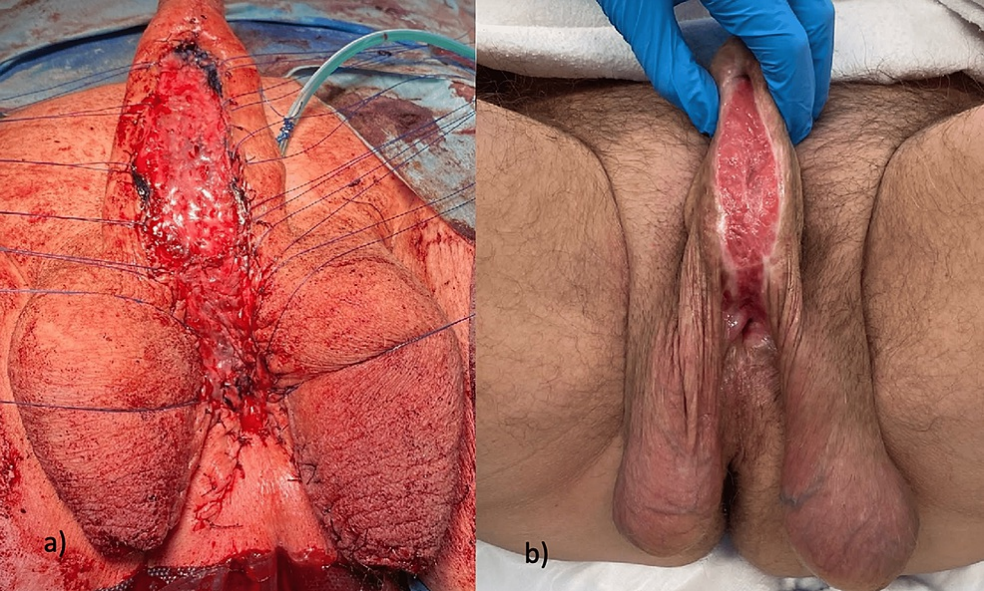
1st stage urethroplasty with T-scrotoplasty a) Intraoperative final result; b) Six months after 1st stage

**Figure 3:**
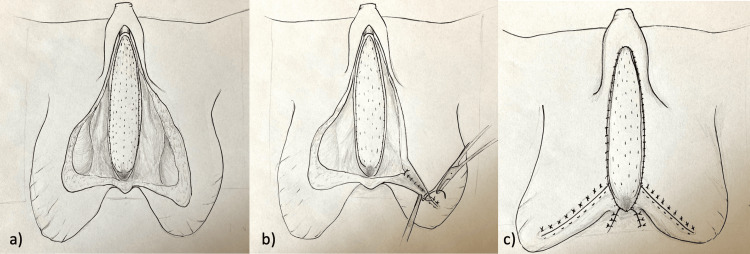
Surgery steps of T-scrotoplasty following the neourethral formation using oral mucosa graft a) The oral graft is fixed on the corpora cavernosa with interrupted quilting sutures (5-0 polyglactin). b) The redundant skin of both scrotal halves is approximated with interrupted sutures (3-0 polyglactin), forming a T-shape at the level of the graft margins. c) The penile skin is adapted to the lateral margins of the oral graft with interrupted sutures (4-0 polyglactin).

The graft was fixed on the corpora cavernosa with quilting sutures (5-0 polyglactin). The lateral margins of the graft were adapted to the penile skin (Figure 2a) using interrupted sutures (4-0 polyglactin). The scrotum had to remain divided to fix the skin to the urethral plate over the entire stricture length. The patient had a large-volume scrotum, so a T-plasty using interrupted sutures (3-0 polyglactin) was necessary for both scrotal halves to allow proper adaptation of the scrotal skin with the urethra without excess skin. Subsequently, the testes were positioned on the left and right sides of the urethra, respectively (Figure 3).

At the proximal, perineal end of the neourethra plate, an inverted U-shaped skin flap was prepared and mobilized to the urethra, creating a Blandy perineal urethrostomy using interrupted sutures (4-0 polyglactin) [7]. An intraoperative cystoscopy showed no evidence of recurrence of a bladder tumor. A 16 French catheter was inserted, and a compression bolster with fatty gauze and tie-over sutures was applied for five days for graft immobilization. The suprapubic catheter already inserted preoperatively was left in situ.

The transurethral catheter could be removed on the 10th postoperative day, and spontaneous micturition was possible via the perineal urethrostomy. The suprapubic catheter was therefore also removed. The patient presented again after six months. The urethral plate was found to be adequately healed with a width of 3 cm, and the tissue was well vascularized, soft, and mobile (Figure 2b).

At this point, the patient reported an unchanged IIEF-5 score of 23, an IPSS score of 2, and an IPPS-QoL of 1 (pleased). Despite his satisfaction with his sexual performance and the relief of his LUTS, he was eager to continue with the second stage of the urethral reconstruction, mainly for aesthetic reasons and his desire to urinate in a standing position. Thus, we proceeded with the second stage repair.

2nd stage

**Figure 4:**
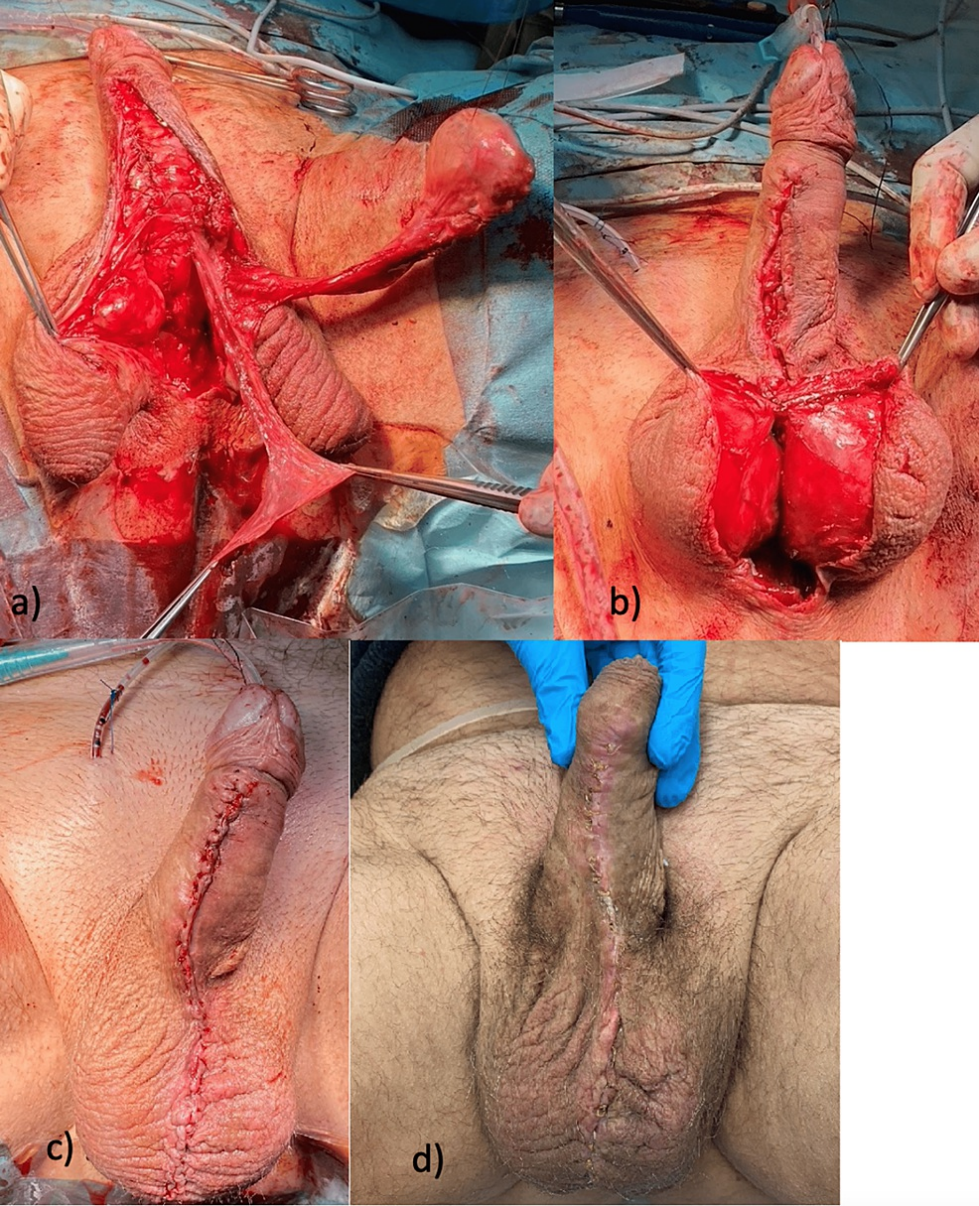
2nd stage urethroplasty with T-plasty release a) Tunica vaginalis flap; b) Scrotoplasty; c) Final intraoperative result; d) Three weeks after the 2nd stage

First, the border between the urethral plate and the skin was sharply incised. Lateral dissection of the skin and dartos fascia was performed. Next, the graft was tubularized in two layers using the first layer of interrupted sutures (5.0 polyglactin) followed by a running horizontal mattress suture (5.0 polyglactin) and a 16 French Foley catheter placement. A pedicled tunica vaginalis flap from the left testis was mobilized and fixed to the anastomosis as an interposition layer (Figure 4a). Subsequently, after loosening the T-plasty, the two hemiscrota were reconstructed into a scrotum using multiple layers of interrupted sutures (3-0 polyglactin) to reapproximate the two halves, and the dartos fascia of the penis and scrotum, as well as the Colles fascia of the perineum, were closed (Figure 4b). Finally, the skin was closed over the entire wound surface (Figure 4c).

Follow-up

Following catheter removal, uroflowmetry showed a bell-formed curve with a maximal flow of 25 ml/sec. The patient reported after two years of follow-up a continued satisfactory erection with an IIEF-5 score of 23 without ventral curvature. The reported IPSS was 3 with an IPSS-QoL of 1 (pleased). In addition, there was no reported postoperative incontinence.

## Discussion

Staged urethroplasty is an excellent technique to treat long-segment, obliterating urethral strictures, especially when complete resection of spongiofibrosis is required [8].

However, depending on the length and location of the stricture, it is a technically demanding procedure that may present different challenges. Depending on the stricture length, an appropriately sized skin flap or oral graft must be selected. Penile skin flaps are often limited in length and width by the existing anatomy [2,9]. Scrotal flaps have the major disadvantage of hairiness, thus should be epilated before being used to form a neourethra [3]. Free buccal grafts can be harvested in a wide range of lengths if the mucosal conditions are healthy. Bilateral buccal augmentations of up to 6-8 cm each can be harvested, and lingual and lower lip grafts are also available and feasible for tissue transfer. Thus, reconstructions up to more than 20 cm are possible [2,10].

Furthermore, the given anatomy must be considered not only when forming the neourethral plate but also during the wound closure. Most publications on staged urethroplasty describe penile strictures, and only a few reports are available on neourethral plate construction and appropriate wound closure at the 1st stage for long-segment strictures extending to the bulbar area [8, 11]. Moreover, all available series of staged urethroplasty including penobulbar strictures do not deal with the issue of wound closure of the scrotum, which can be occasionally complicated, mainly in cases of excess skin. In the review article of Mori and Angermeier, only one photo of a penobulbar staged procedure is presented, with a relatively small scrotum sutured sideways with the neourethra without any ancillary procedures [1]. However, there are no reports on the reconstructive closure of large-volume scroti or the performance of a scrotoplasty in the setting of staged urethroplasty.

In our case, an ancillary T-shaped scrotoplasty had to be performed in the 1st stage to be able to anastomose the long scrotal skin surface bilaterally with the neourethral plate. Alternatively, part of the excess skin of both hemiscrota could have been trimmed tangentially to allow a simple side-to-side anastomosis with the neourethra. However, this would have resulted in altered scrotum anatomy after reapproximating in the 2nd stage. Considering a possible patient's dissatisfaction with a smaller scrotum due to the surgery, the described T-shaped scrotoplasty offers the possibility of leaving the appearance of the scrotum unchanged in the long term. Furthermore, moving the scrotum away from the midline offers the additional advantage of better graft take since excess scrotal tissue interposition would compromise the effectiveness of the applied compression bolster and the tie-over sutures.

The issue of scrotal reconstruction would not be of concern in the case of a single-stage urethroplasty by using a penile invagination into the perineum according to the Kulkarni technique, requiring a much smaller single perineal skin incision [5]. Regarding the absence of an adequate native urethral plate, a solitary one-side augmentation technique for long segment strictures, such as the dorsal onlay graft technique described by Barbagli et al. [12], the dorsolateral onlay graft technique described by Kulkarni et al. [13] or the penile fasciocutaneous skin flap described by McAninch and Morey [14], would not be sufficient to create a 28 French urethral patency.

The combined technique of Joshi et al. using buccal graft dorsally plus fasciocutaneous penile flap ventrally could be a feasible option, augmenting the urethra up to a 28.6 French caliper, however, requiring an additional circumcising subcoronal incision. He reported in a series of 15 patients a primary success rate of 86.7%, defined as no need for any kind of instrumentation, while the secondary success rate was 73.3%, defined as a Qmax under 10 ml/s or new obstructive LUTS [15]. Although we occasionally use Joshi's technique, in this case, given the severe spongiofibrosis and compromised tissue vascularization, we preferred a staged technique to maximize the chances of graft take.

Limitations of the current technique include the usual complications of every staged urethroplasty, especially following second-stage tubularization including urethrocutaneous fistula, wound dehiscence, and recurrent stricture. Thus, these risks should be addressed by the urologists in the framework of a detailed preoperative written consent process.

To the best of our knowledge, the T-shaped scrotoplasty technique has not been described so far and resulted in an excellent functional outcome for our patient and a satisfactory cosmetic outcome following closure.

## Conclusions

In our case, a successful two-stage urethroplasty was performed for a stricture length of 15 cm. The neourethral plate was constructed with free oral mucosa grafts. The major challenge was a large-volume scrotum. Following cleavage of the scrotal sac and a T-plasty, the two hemiscrota were anastomosed to the neourethral plate without difficulty in the first session. In the second stage, tubularization of the urethra and skin closure with the reconstruction of the scrotum by releasing the T-plasty was performed without problems. The presented T-scrotoplasty technique allows individual scrotal skin placement for anastomosis to the neourethral plate with very good cosmetic and functional results.
